# Editorial Department

**Published:** 1874-04-01

**Authors:** 


### Editorial Department.

HAVE WE ANY CHOLAGOGUES?—It seems to us that the author of the essay on this question, in the present number of THE EXAMINER, like most others who have experimented on the same subject, has made a mistake in looking for cholagogues among cathartics, or in regarding the active cathartic action of any medicine as a test of its power to increase the secretions of the liver, or of any other organ, except the follicles of the mucous membrane of the intestines. We learned, by simple clinical observation, many years since, that when any medicine was given in sufficient doses to act directly on the bowels as a cathartic in a few hours, it had little or no effect in increasing the action of any other organs. Indeed, few facts are more familiar than that profuse intestinal discharges, whether from cathartics or spontaneous diarrhœa, speedily cause a diminution both in the secretions of urine and in the evidences of bile in the evacuations. Hence, to test the action of any medicine on the liver, or any other secreting organ outside of the mucous membrane of the alimentary canal, it should be given in such a way as not to induce co-incident active evacuations in any other direction.

PROFESSIONAL CONFIDENCE.—The *Times* newspaper of this city has recently made a persistent effort to create the impression that Professor H. A. Johnson, of this city, in an article published in the *Tribune*, March 3d, had grossly violated professional honor, and betrayed the sacredness of the relation between physician and patient, by using the following expression: "He (Storey) charges me with having withheld important facts from the profession. The only *fact of which I am aware, bearing on the case, and not stated, was, that she* (Mrs. Storey) *had been a prostitute.*" To make the allegation of this fact appear as a breach of professional confidence, it must be assumed that his knowledge of the fact was obtained by professional intercourse with the lady, or her husband. But the truth is, that not a word had ever passed between the doctor and his patient concerning *prostitution*, or any of its consequences. And the foregoing allegation, made in self-defence, was founded entirely on knowledge obtained from *public sources*, and was in no sense a betrayal of the confidential relations between the physician and patient.

AMERICAN MEDICAL ASSOCIATION.—This important national organization is to hold its next annual meeting in Detroit, Michigan, commencing on Tuesday, *June* 2d, 1874. We call special attention to this date, lest some might forget, as the time of meeting has more frequently been the first Tuesday in May. But the northern location of Detroit makes the first Tuesday in *June* much the more pleasant time for such a gathering. We trust the profession throughout the North-Western States will appreciate the necessity of contributing its full share, both to the numbers in attendance and the interest of the occasion. There are some important constitutional amendments pending, to be acted upon at that meeting. Their object is, to make the Association a more directly representative body, by limiting the representation to State, District, and County medical societies, and cutting off all schools, hospitals, and other medical organizations. The proposition is an important one, and should be well considered.

